# Supplementary figures and images for: Regulation of host gene expression by J paramyxovirus
Source: PLoS One. 2023 Nov 14;18(11):e0294173. doi: 10.1371/journal.pone.0294173 (PMC10645344; doi:10.1371/journal.pone.0294173)

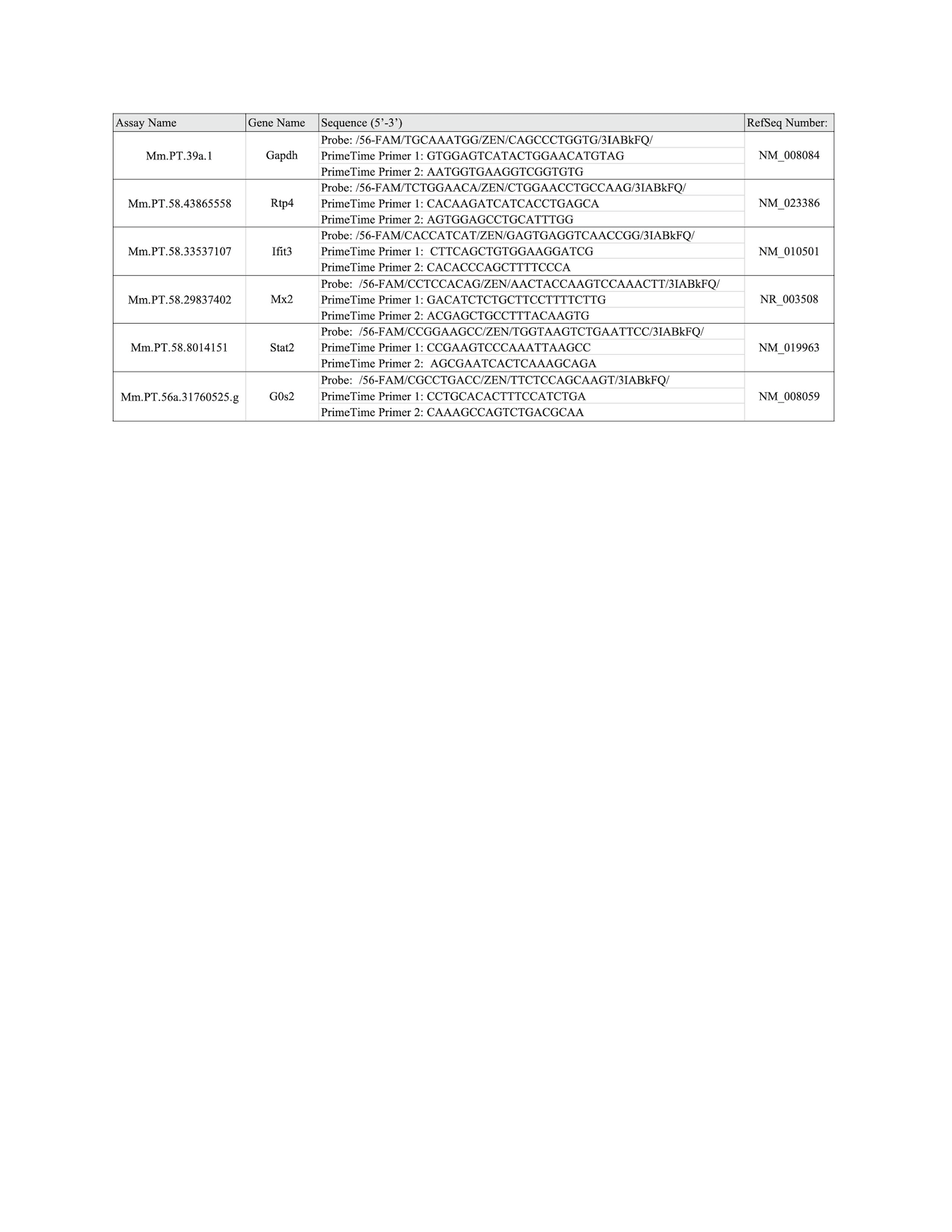

Supplement: S1 Table — (TIF) [file pone.0294173.s001.tif]

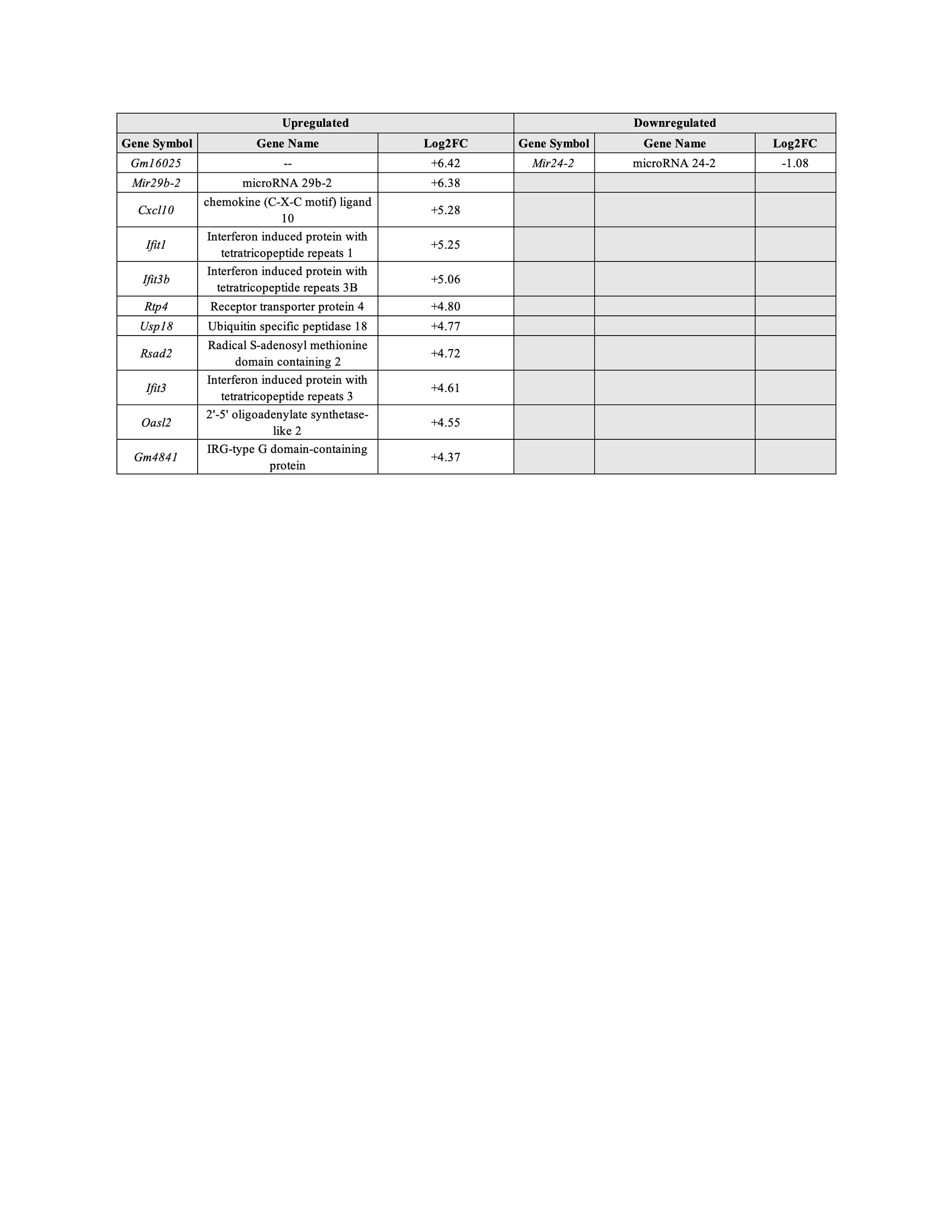

Supplement: S2 Table — (TIF) [file pone.0294173.s002.tif]

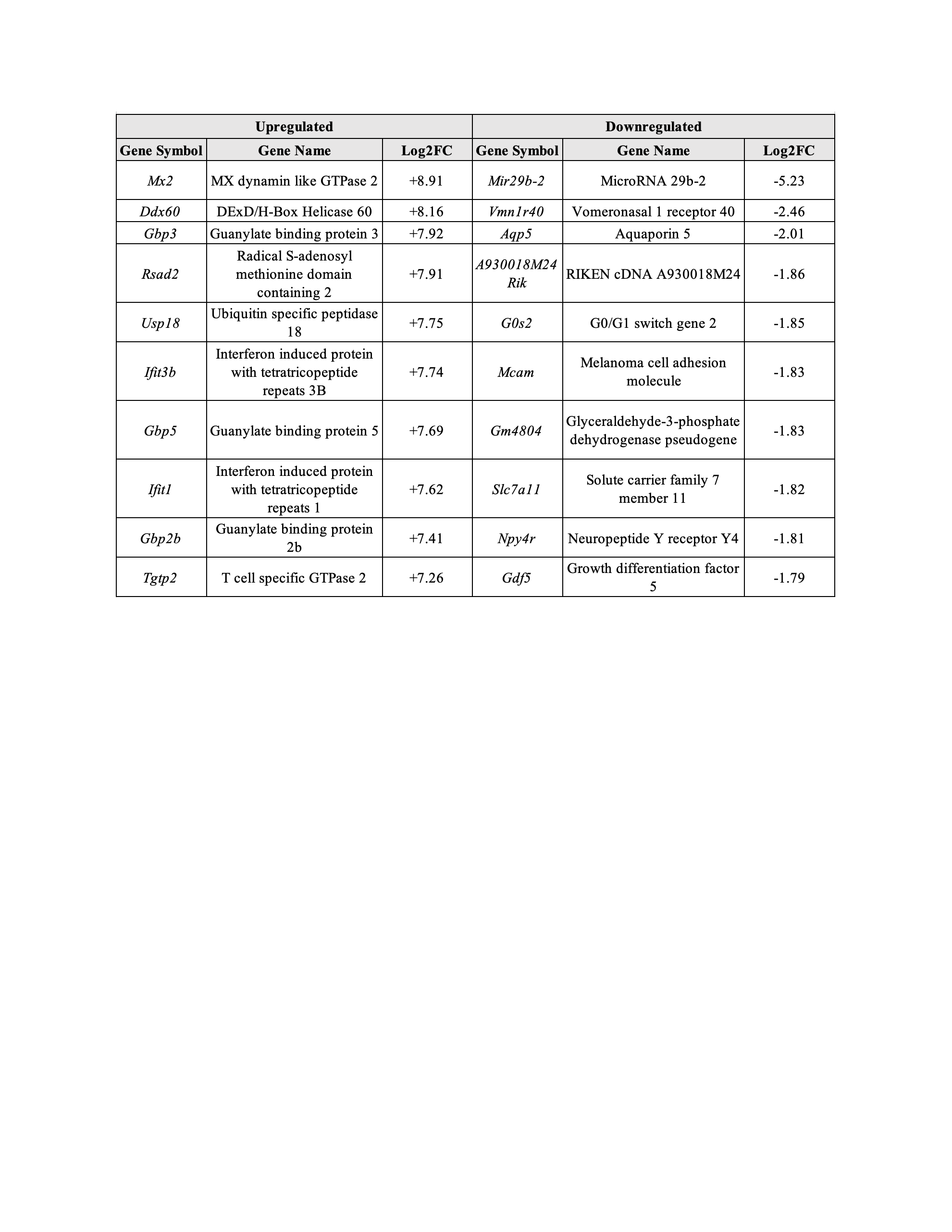

Supplement: S3 Table — (TIF) [file pone.0294173.s003.tif]

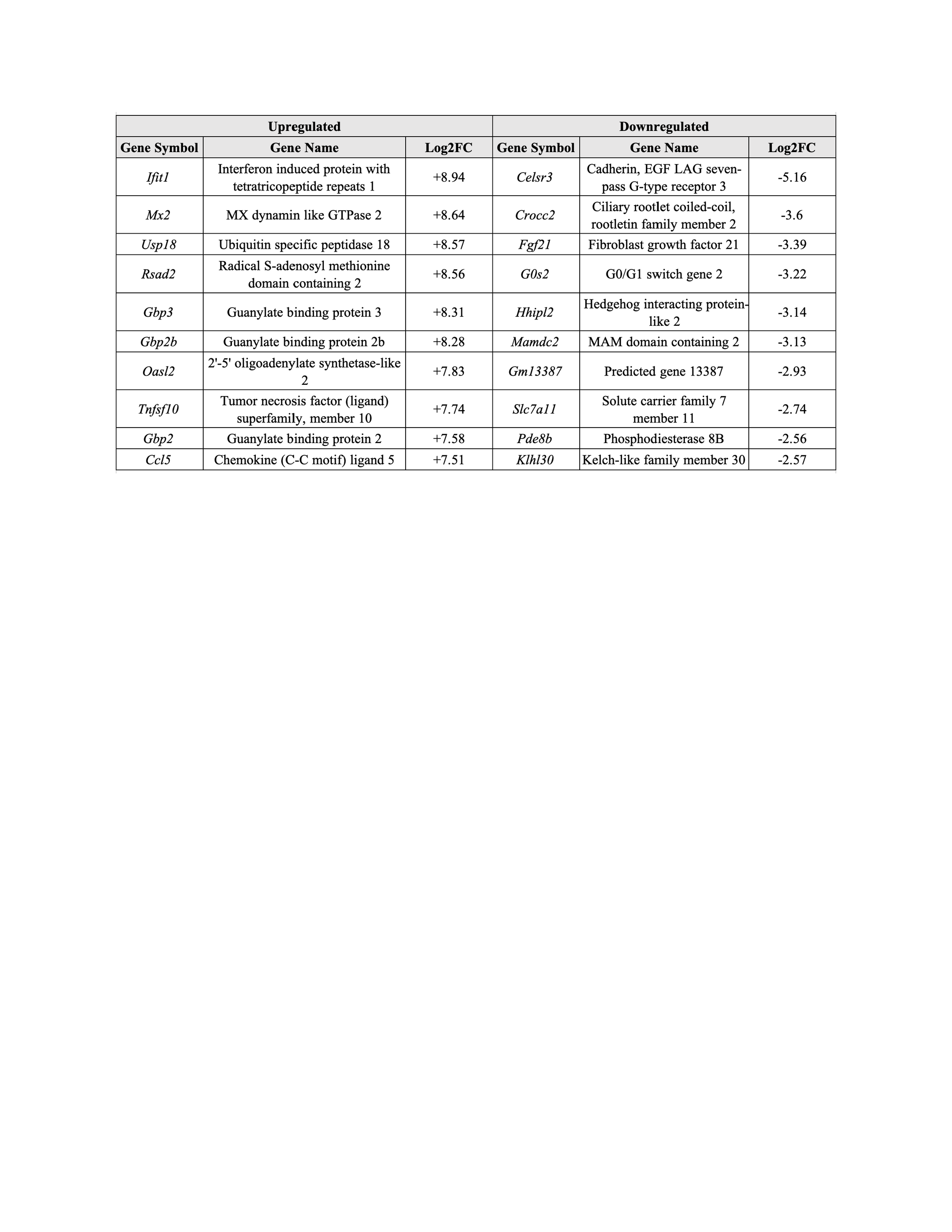

Supplement: S4 Table — (TIF) [file pone.0294173.s004.tif]

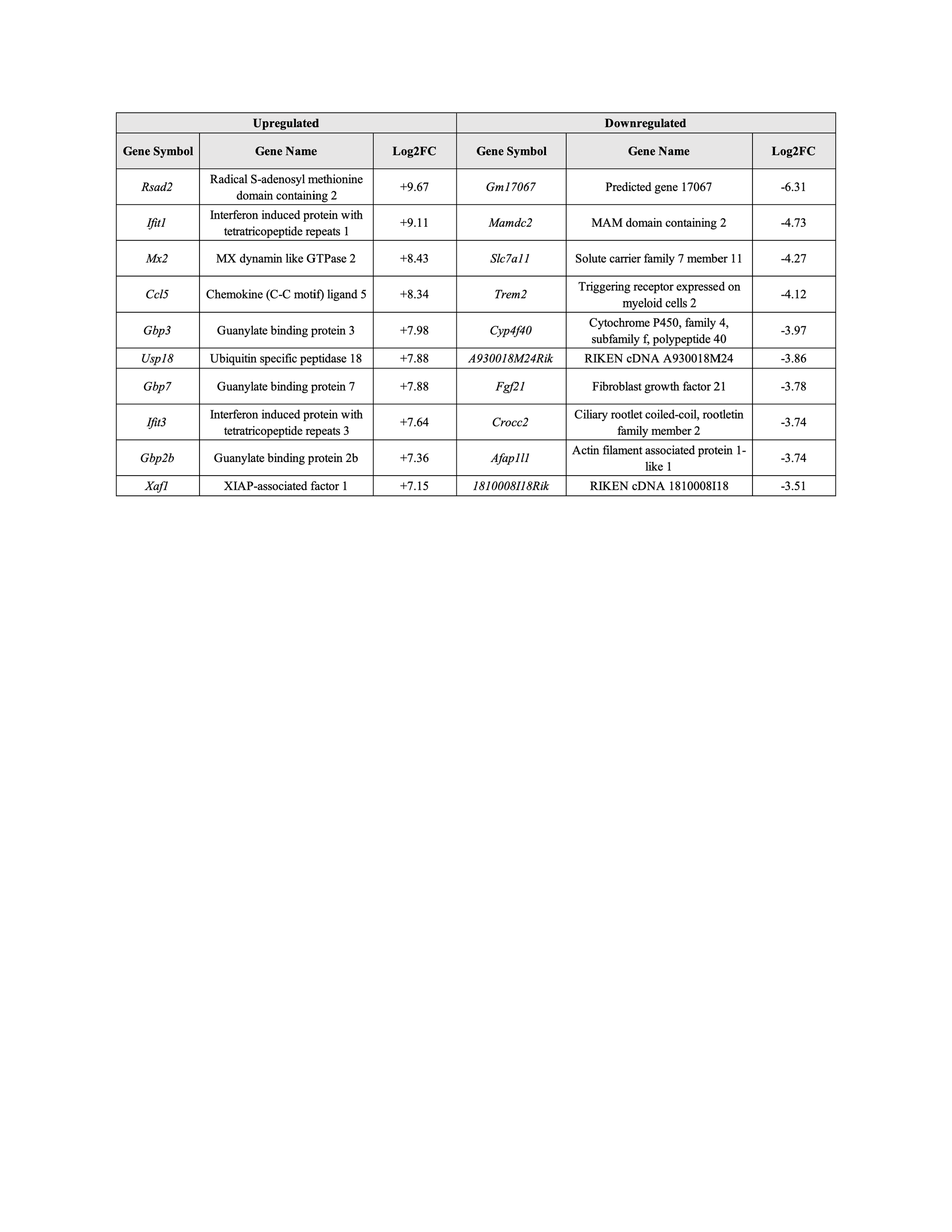

Supplement: S5 Table — (TIF) [file pone.0294173.s005.tif]

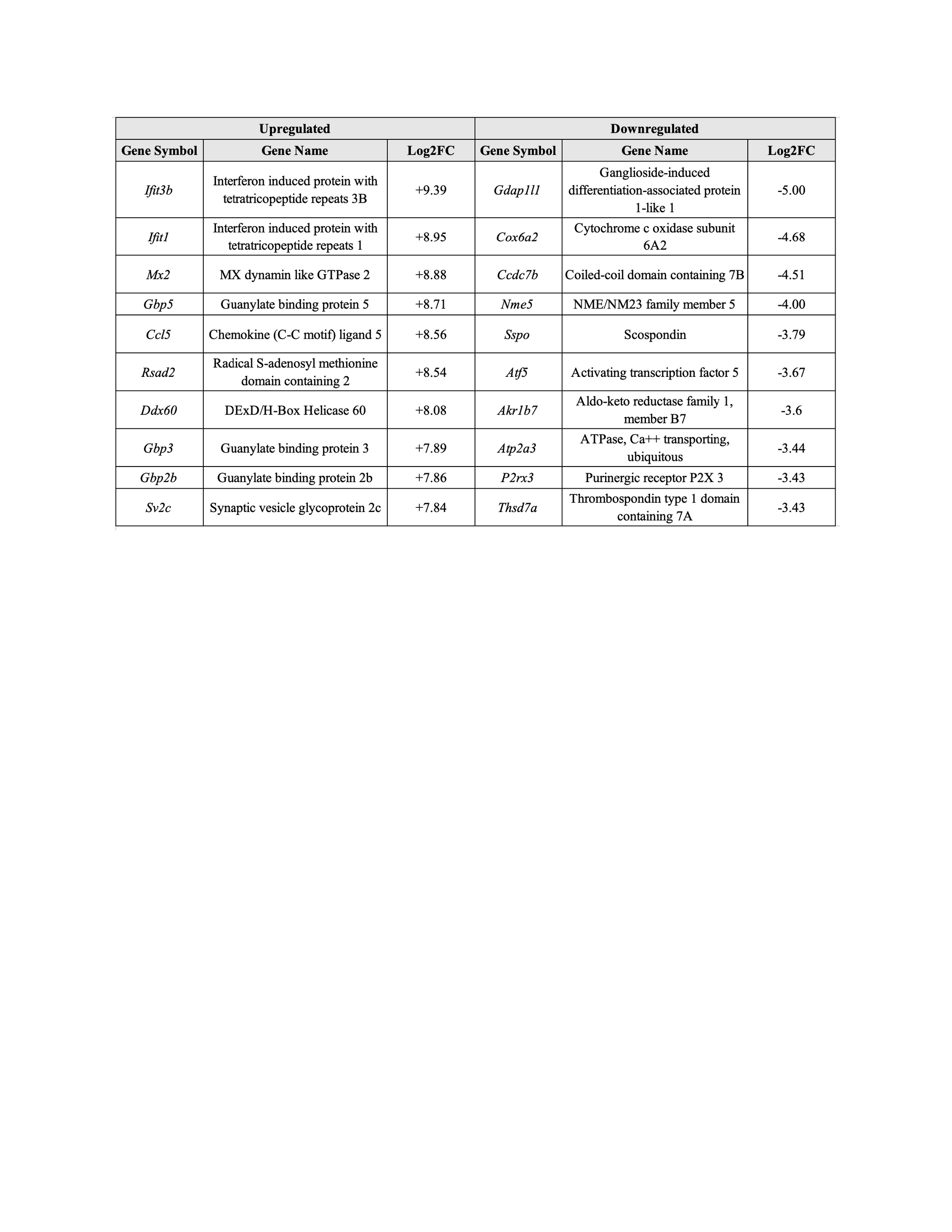

Supplement: S6 Table — (TIF) [file pone.0294173.s006.tif]

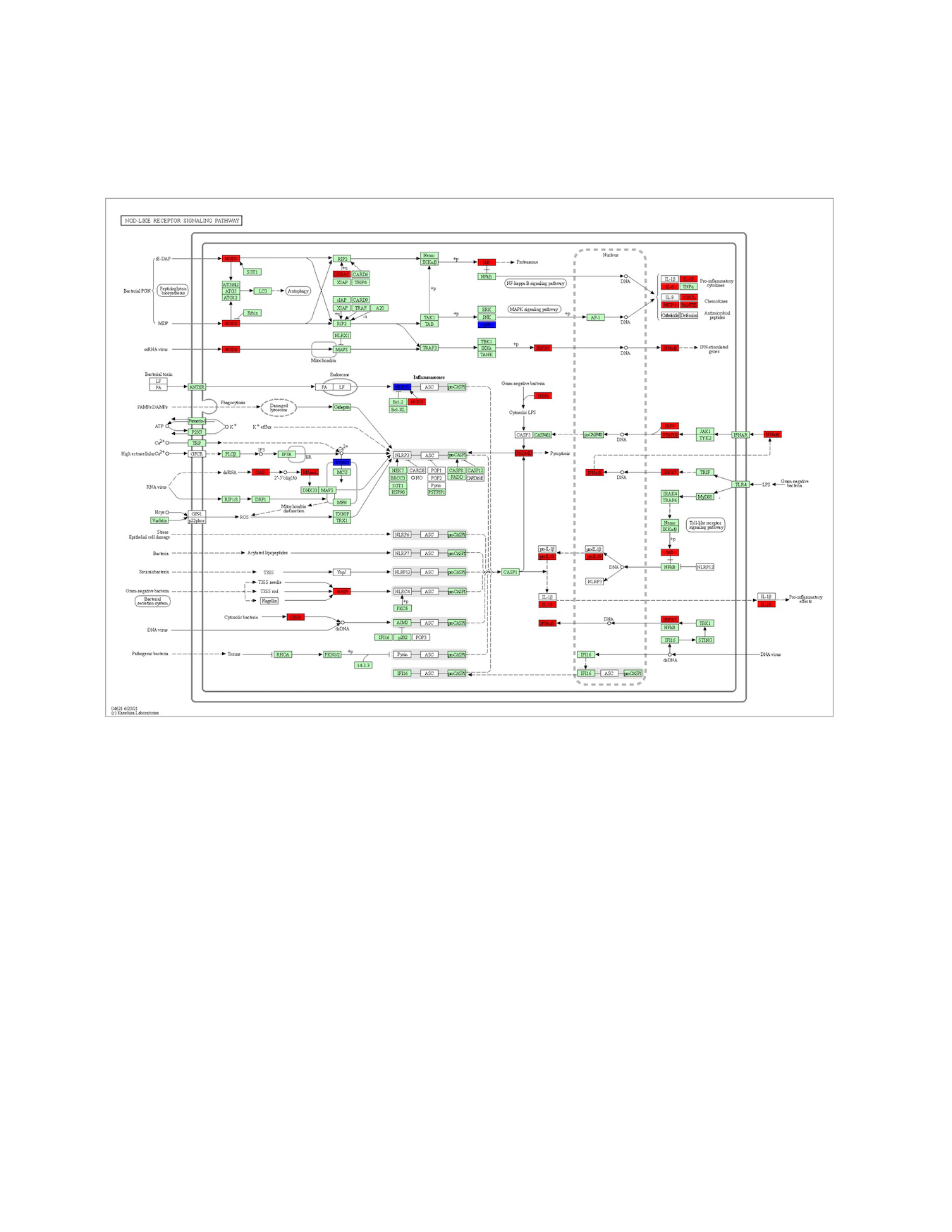

Supplement: S1 Fig — Red and blue shading indicates increased and decreased expression, respectively, in JPV-infected cells relative to the mock-infected cells. White and green shading indicates non-expression and non-differential expression, respectively. Solid and dashed lines represent direct and indirect interactions, respectively. (TIF) [file pone.0294173.s007.tif]

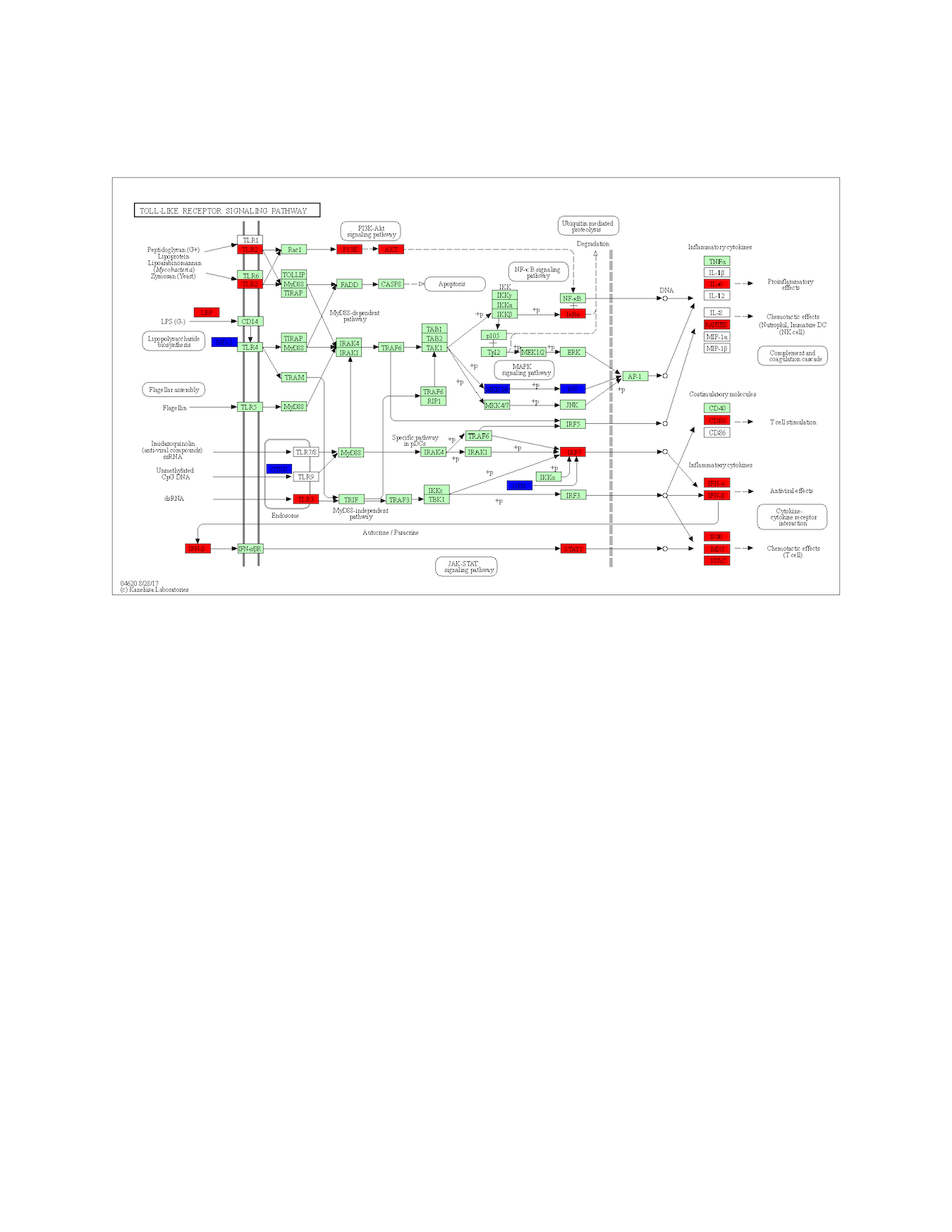

Supplement: S2 Fig — Red and blue shading indicates increased and decreased expression, respectively, in JPV-infected cells relative to the mock-infected cells. White and green shading indicates non-expression and non-differential expression, respectively. Solid and dashed lines represent direct and indirect interactions, respectively. (TIF) [file pone.0294173.s008.tif]

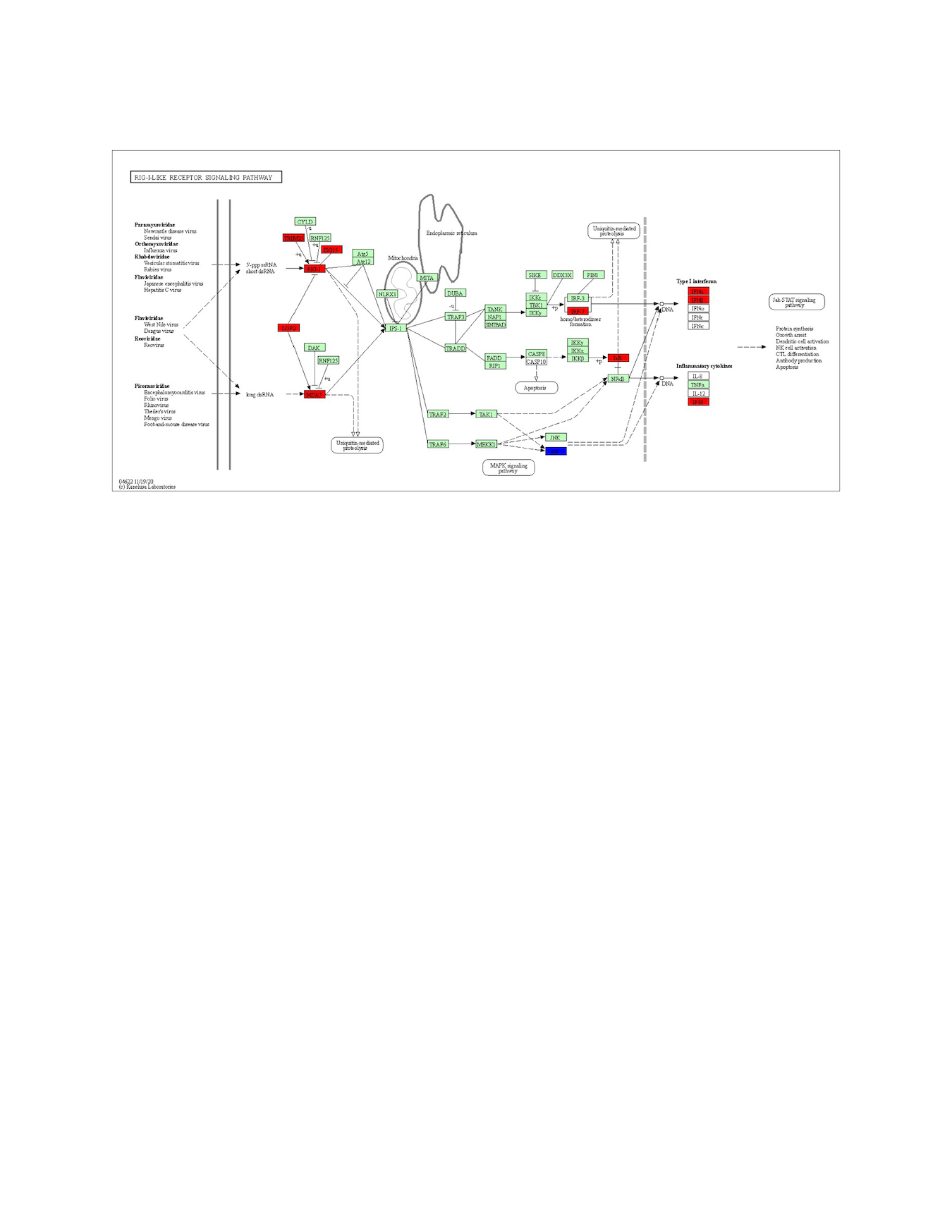

Supplement: S3 Fig — Red and blue shading indicates increased and decreased expression, respectively, in JPV-infected cells relative to the mock-infected cells. White and green shading indicates non-expression and non-differential expression, respectively. Solid and dashed lines represent direct and indirect interactions, respectively. (TIF) [file pone.0294173.s009.tif]

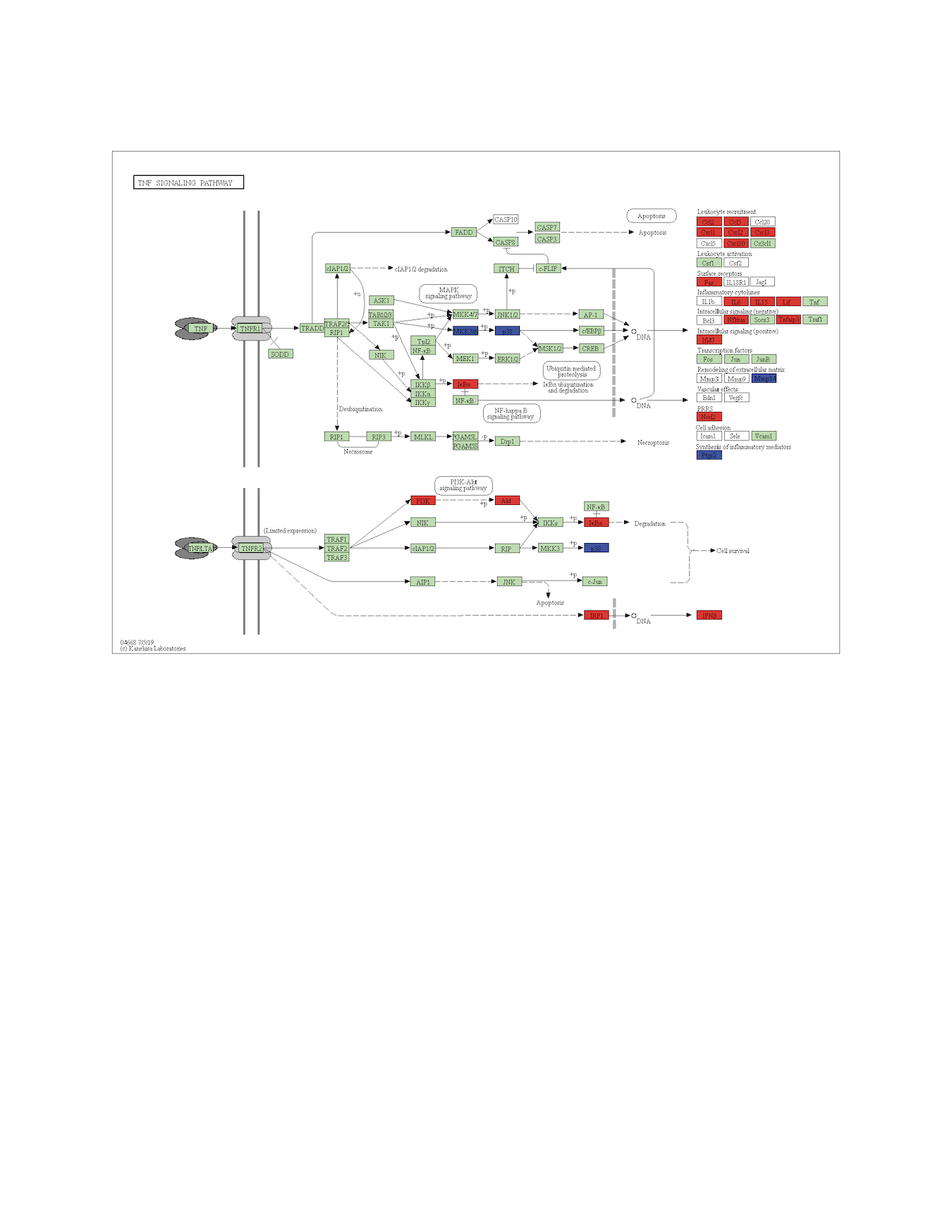

Supplement: S4 Fig — Red and blue shading indicates increased and decreased expression, respectively, in JPV-infected cells relative to the mock-infected cells. White and green shading indicates non-expression and non-differential expression, respectively. Solid and dashed lines represent direct and indirect interactions, respectively. (TIF) [file pone.0294173.s010.tif]

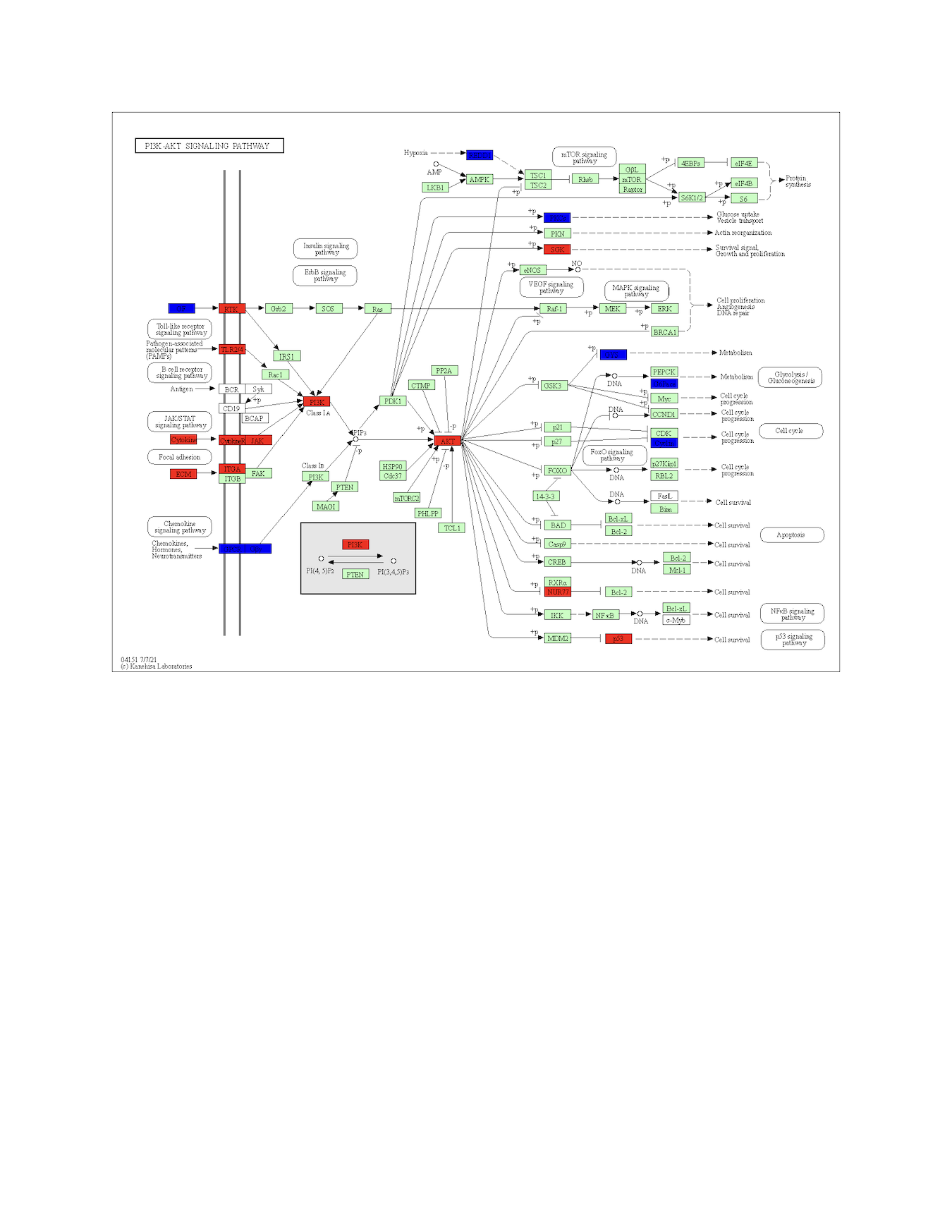

Supplement: S5 Fig — Red and blue shading indicates increased and decreased expression, respectively, in rJPV-infected cells relative to the mock-infected cells. White and green shading indicates non-expression and non-differential expression, respectively. Solid and dashed lines represent direct and indirect interactions, respectively. (TIF) [file pone.0294173.s011.tif]
