# Supplementary material for: Regulation of host gene expression by J paramyxovirus
Source: PLoS One. 2023 Nov 14;18(11):e0294173. doi: 10.1371/journal.pone.0294173 (PMC10645344; doi:10.1371/journal.pone.0294173)
Supplement: Attachment — Submitted filename: Response to Reviewers V1.1.docx [file pone.0294173.s012.docx]

**Responses to Reviewers**

**Reviewer #1:**

1. This manuscript contains a lot of acronyms and jargon that are not always explained and/or spelled out. It would help with the readability of this manuscript if this issue was resolved throughout the manuscript.
2. Line 65: define what F and G are. **–**

**Acronyms are now defined.** **Functions of F & G are further defined in lines 87-89.**

3. Line 117: What was used for mock infection?

**L929 cells that were incubated with infection media that had no virus. After the 1hr incubation at 37^0^C, the mock cells were washed with PBS and had maintenance media added back. This was the same treatment as the virus-infected cells.**

4. Line 119: What is P/S?

**1% penicillin-streptomycin (P/S), defined in lines 107-108 of the manuscript.**

5. Line 147: Where is this GACRC located? At UGA?

**Yes, it is at UGA – this was added in the manuscript at line 149.**

6. Also, the figure labels are very small. There seems to be plenty of space to make the font larger and more readable.

**The figure labels are sized based on PLOS ONE manuscript guidelines.**

**Reviewer #2:**
1. Seven time points were designed after infection, while 48 h samples were more focused. I would suggest combining all samples and concentrate on some common features, such as genes showing significant changes in all time points (or most time points). In addition, how about the determination of endpoint at 48 h, could longer duration lead to further immune responses?

**One purpose of this study was to examine differences in host gene expression across different timepoints during infection and to examine when certain host genes/pathways are triggered as JPV replicated. Thus, we feel it is important to keep data from each timepoint separated.** **However, in the revised manuscript, we have included a table that lists DEGs that are shared across most/all of the timepoints. When infecting with a high MOI, JPV titers reach the highest point around 48hpi. That is why we chose to examine changes in gene expression up until 48hpi because we wanted to see what genes are up- or down-regulated as viral replication increases exponentially. Further studies could examine changes in gene expression after 48hpi as viral titers decrease, but the differences in the host transcriptome may not be as pronounced. Alternatively, future studies could be done with a low MOI and examine differences in host gene expression with JPV replicating at a different rate.**

2. There are some grammatical errors to be checked.
There should be space between number and hpi.

**Fixed in the manuscript & in the figures.**

In the legend of Table 6. “Table 6” appeared for twice.

**Fixed**

**Reviewer #3:**

The abstract has technical details (e.g. the name of the sequencer and the name of the software) that take up space that would be better used for more relevant information, like the length of reads and whether paired-end or not. The authors should be more explicit about the length of the reads and whether paired-end or not. It seems that “SE75” (line 143) means single end reads of 75 nt but that is not completely clear.

**SE75 means single-end 75 base paired sequencing, which is now denoted in the manuscript at line 146. We have now included this information in the abstract at line 38.**

The tables are a challenge of readers. Table 1 and Table 2 would be better placed with the Methods or as Supplementary Materials. This is important information but it is not directly pertinent to the main thrust of the report. The results in Table 3 could be presented in the text. Tables 4-10 repeat a lot of the same information. For example, why does Mx2 need to be defined in more than one table? The authors are advised to present this information in a more concise and readable form. Perhaps there could be summary table in main text and the extensive details for each time point in supplementary materials.

**In these types of studies, the summary of sequencing data and the # of total DEGs detected at each timepoints is typically included in the results section. Thus, we feel it is important to keep Table 1, 2, and 3 in the results section. Table 3 is necessary for readers to easily see how many DEGs were found at each timepoint and show the dramatic increase in gene expression as infection progresses. The purpose of Tables 4-10 was to demonstrate at which timepoint certain immune-related genes started to be upregulated. We will include table 4 for 2hpi, and table 10 for 48hpi in the main manuscript to really highlight these differences and move the other tables to the supplementary materials.**

This was a single experiment with one strain of virus and one type of cell line. There could be more discussion of limitations of the study, such as the dependence on one lineage of mouse fibroblast cells. Transformed cells likely have lost the capacity for expression of some genes that would be relevant to infections of whole animals or primary cultures of fibroblasts. This does not mean that further experimentation is called for, but readers should be informed.

**Added in** **from lines 490-496:** “**In this study, we have attempted to provide a comprehensive overview of the transcriptomic changes during JPV infection in L929 cells. A limitation of this study was the use a single immortalized mouse cell line. It is possible that the transformed cells have lost the ability to express certain genes that mediate JPV infection. Thus, future studies examining gene expression during JPV infection in both primary fibroblast cultures and *in vivo* are critical to obtain a complete understanding of the host-pathogen interactions of jeilongviruses.”**

Figure 5, which is large and just copies of pathways obtained from other sources. Much of this is not relevant here. Figure 5 could be deleted.

**Similar studies examining changes in cellular gene expression during viral infection have included these types of figures. These pathways were taken from the KEGG pathway database, which we used to show which genes in immune-related pathways are differentially expressed during JPV infection at 48 hpi. Thus, we feel it is important to include these pathways as a main figure of the paper, like other studies have done.**

The findings from this single experiment are important addition to the literature on this particular virus but were pretty much what would be expected in terms of responses to a RNA virus. The discussion could have devoted more space to a comparison with what has been found in other studies of virus infection of transformed fibroblast cells instead of dwelling so much on descriptions of what different genes and pathways do.

**We have updated the discussion section to compare what we found to other studies examining host genes/pathways in RNA viral infection.**

I understand that this is a descriptive study without a clearly defined hypothesis but the authors could help the reader better understand what the bigger picture is here.

**Added in lines 103-107 of manuscript: “Given that JPV induces cytopathic effects *in vitro*, we hypothesize that JPV infection will induce significant changes in the cellular transcriptional response as the virus replicates, and that we will be able to quantify these differences using RNAseq. These results will provide us with a detailed overview of the changes in host gene expression caused by jeilongvirus infection.”**

I saw no mention of the public availability of the raw data, such deposit of the reads with SRA. There should also be an associated BioProject and BioSamples or the equivalent of these. This is essential.

**The raw data have been already uploaded to** NCBI SRA (sequence read archive) database <https://www.ncbi.nlm.nih.gov/sra> and **will be publicly available on publication of our manuscript.**
