# Supplementary material for: Regulation of host gene expression by J paramyxovirus
Source: PLoS One. 2023 Nov 14;18(11):e0294173. doi: 10.1371/journal.pone.0294173 (PMC10645344; doi:10.1371/journal.pone.0294173)
Supplement: Attachment — Submitted filename: RTR Rev2.1.docx [file pone.0294173.s013.docx]

**Responses to Reviewers**

**Reviewer #2:**

**The authors state "in the revised manuscript, we have included a table that lists DEGs that are shared across most/all of the timepoints", could you specify which table as I did not find it?**

In place of that proposed table, we decided to include a table showing the top DEGs at 2 and 48hpi in the main manuscript. This will show the differences between the earliest and latest timepoint. The other timepoint tables have been moved to supplemental. We discuss commonly shared DEGs across the timepoints in the manuscript.

**Reviewer #3:**

**The authors have submitted an improved manuscript. But I was not persuaded by the argument for including four figures of pathways. This may be done in other papers but that doesn't mean that it is justified here. There is just too little experimental data here for these sorts of conclusions. These figures may be okay in supplementary material but not in main article.**

We have moved all pathway figures to the supplemental materials. We have removed 2 of the pathway figures. Now there are 5 pathway figures in supplemental instead of 7.

**Minor point:**

**There are too many significant figures in the tables. It is highly unlikely that the measurements were so accurate--out to severe decimal points.**

We changed to log2FC to have only 2 decimal points.
